# Supplementary material for: Exploring a novel genomic safe-haven site in the human pathogenic mould Aspergillus fumigatus
Source: Fungal Genet Biol. Author manuscript; Available in PMC 2025 Jun 22. (PMC7617788; doi:10.1016/j.fgb.2022.103702)
Supplement: Supplementary Material [file EMS206535-supplement-Supplementary_Material.pdf]

SUPPLEMENTARY DATA

Supplementary Table1: Oligonucleotide used in this study.

Supplementary Table2: Characteristic features of the identified genetic safe-haven sites in *A. fumigatus*.

Supplementary Table3: Detailed characteristics of the SH-*aft4* in clinical and environmental *A. fumigatus* isolates

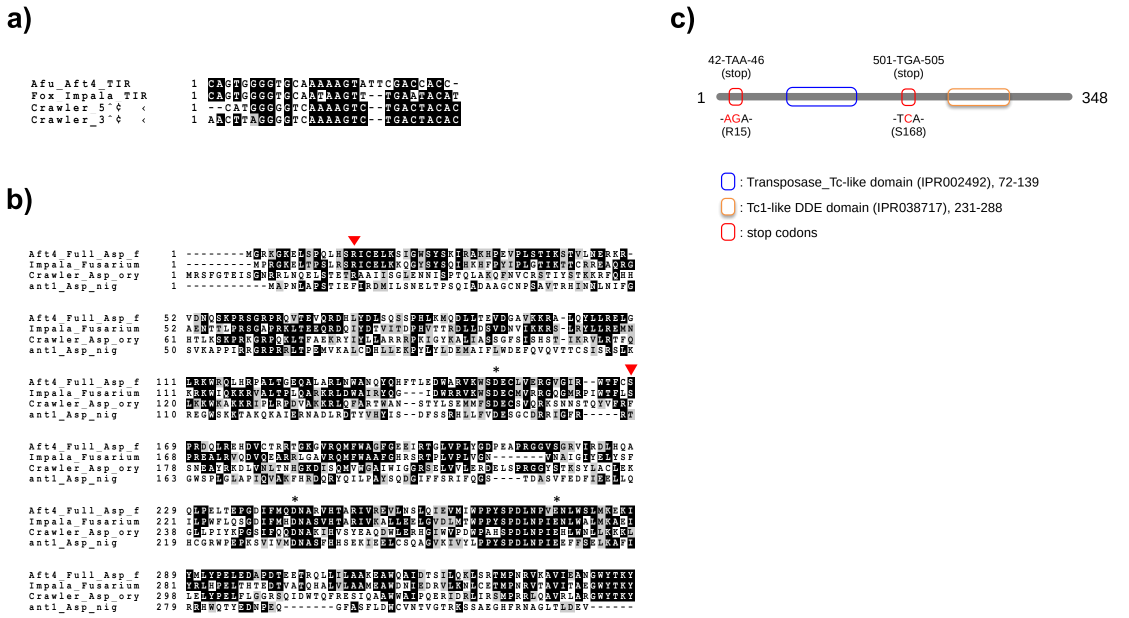

Supplementary Figure 1: Domain structure and sequence alignment of the putative full-length form of Aft4 transposable element. (a) Alignment of Terminal inverted

repeat (TIR) sequences of *A. fumigatus aft4*, *Fusarium oxysporum impala*, and *A. oryzae Crawler*. **(b)** Schematic representation of the domain structure of the putative full-length form of the *A. fumigatus* Aft4 transposase. The transposase Tc-like domain (IPR002492), and Tc1-like DDE catalytic domain (IPR038717) predicted by InterPro Scan is shown in blue and orange, respectively. The position of the stop codons found within the putative full-length ORF of *aft4* are shown in red with possible original codons. **(c)** Multiple protein sequence alignment of the transposase of the full-length form of the *A. fumigatus* Aft4, *F. oxysporum impala* (Langin et al. 1995), *A. oryzae Crawler* (Ogasawara et al. 2009)*, and A niger* Ant1 (Glayzer et al. 1995). The conserved amino acids consisting of the DD(35)E catalytic triad are indicated by asterisks. The position of the stop codons found in the inactivated *aft4* ORF are indicated by red triangles.

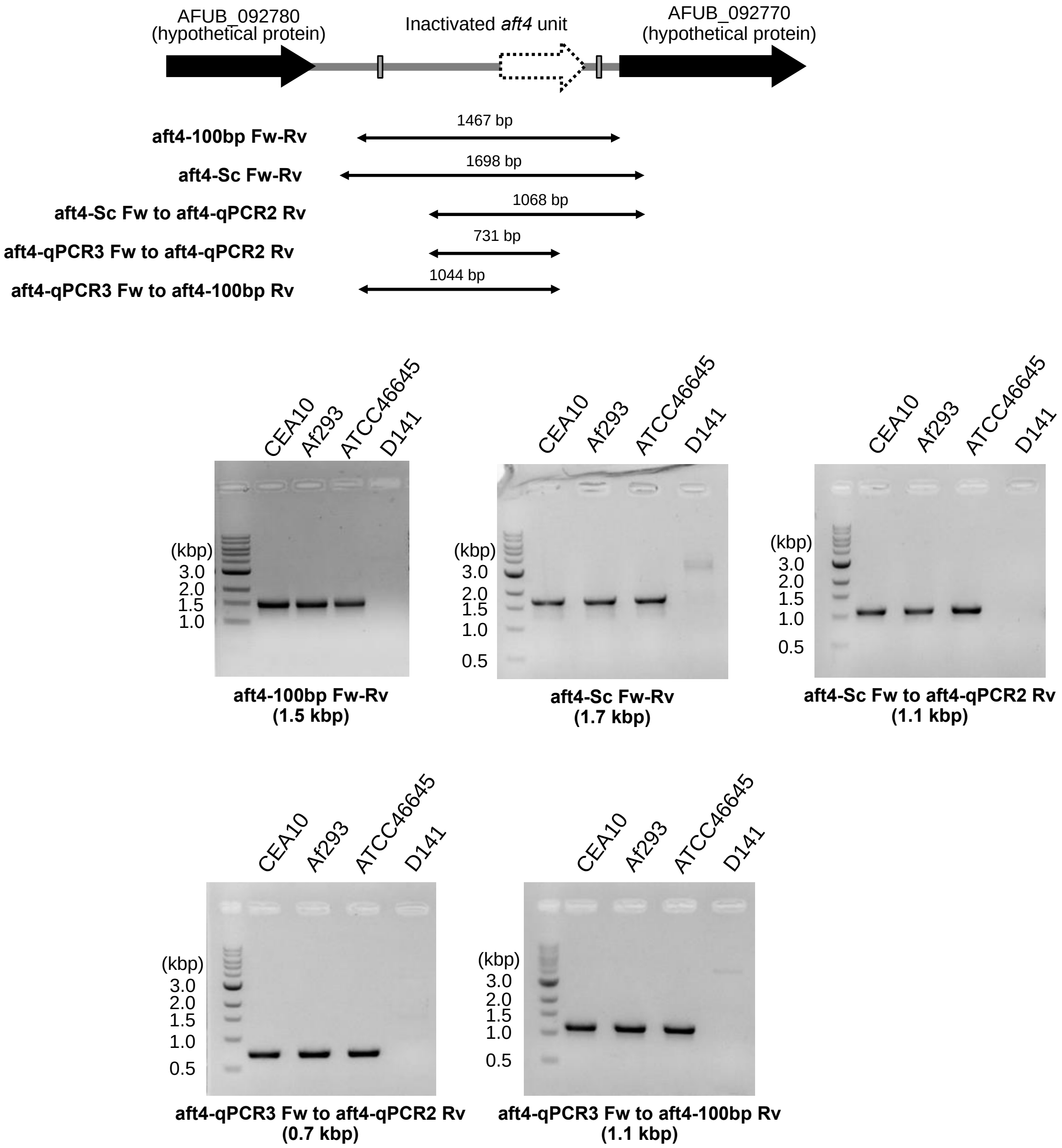

Supplementary Figure 2: Diagnostic PCR analysis revealing the presence of the *aft4* locus in the genome of the *A. fumigatus* isolates in common laboratory use. (a) Primers used in the diagnostic PCR analysis are shown with the expected size of the PCR product, which is estimated from the *A. fumigatus* A1163 genome. **(b)** Results of the diagnostic PCR analysis. 10 ng of genomic DNA was used as a template for each PCR reaction and the generated PCR products were resolved on a 1% agarose gel. Positive amplification of the putative inactivated *aft4* ORF and the 1.7-kb region containing the

ORF with 200-bp of the flanking sequences were observed for *A. fumigatus* Af293, CEA10 and ATCC46645.

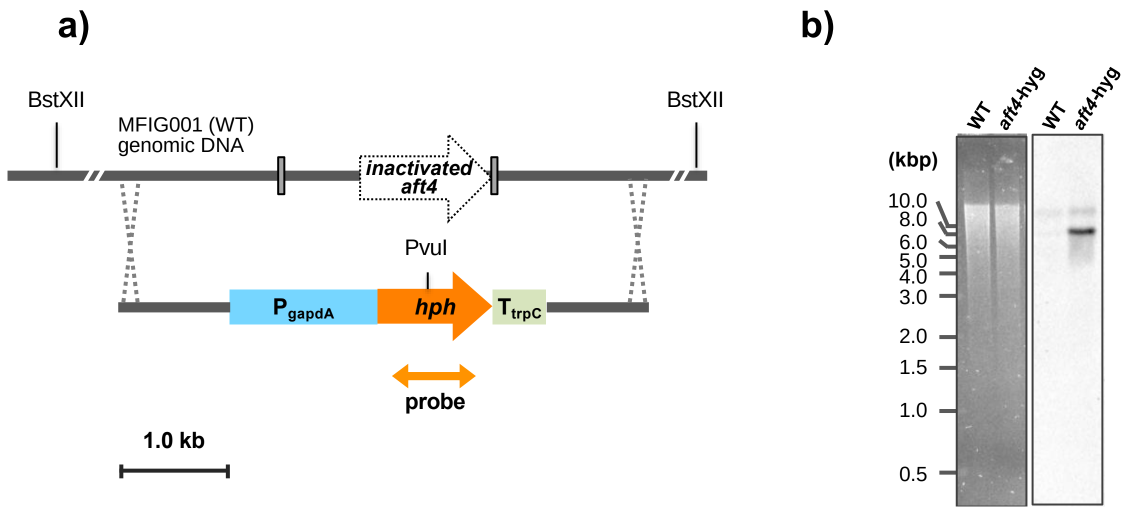

Supplementary Figure 3: Southern blotting analysis verifying single copy integration of the hygromycine resistance (*hph*) cassette at the *aft4* locus. (a) Schematic representation of the construction of the *aft4* knockout mutant (*aft4-hyg*). The gene replacement cassette was constructed by fusion PCR and introduced into the wild-type *A. fumigatus* MFIG001 via homologous recombination. **(b)** Southern blot analysis of the *aft4-hyg* mutant. The restriction enzyme and the probe used in the Southern blot analysis are shown in the figure.

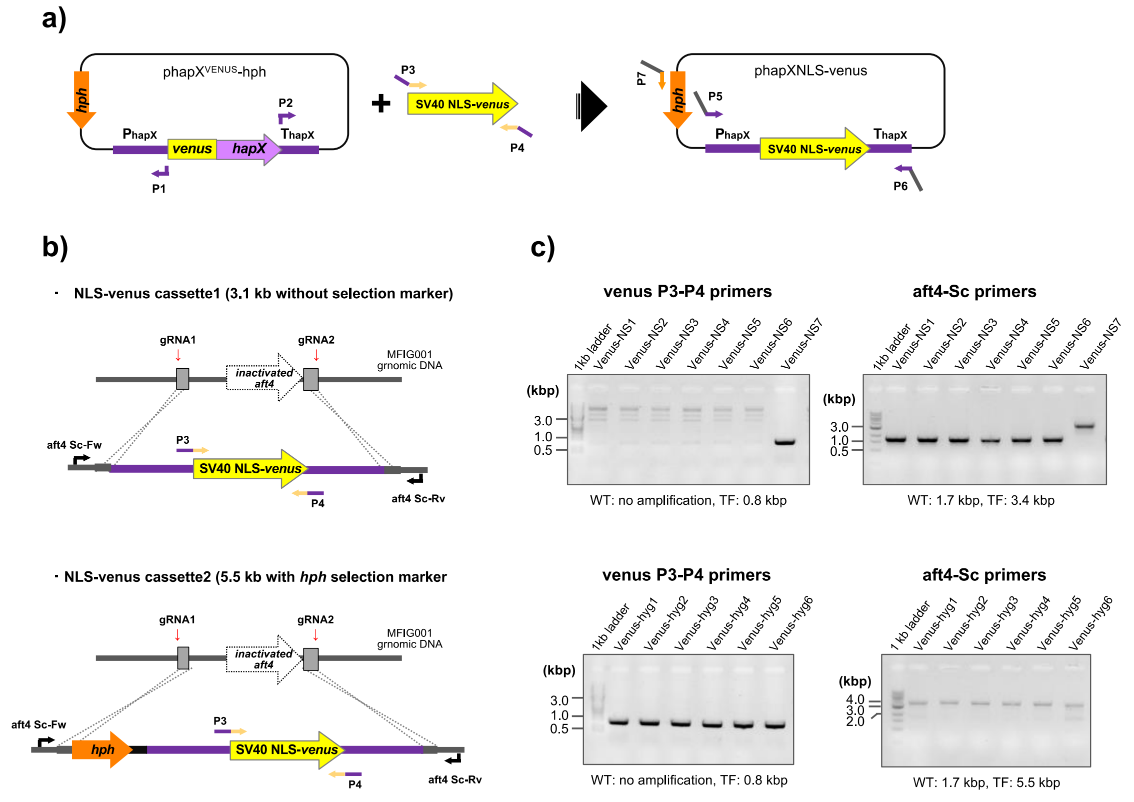

Supplementary Figure 4: Construction of the NLS-Venus expressing mutants. **(a)** Scheme of the construction of NLS-venus expression cassettes for CRISPR-Cas9 mediated transformation. A DNA fragment containing 1.2 kb of the 5′- and 1 kb of the 3′-flanking regions of *hapX* derived from phapX^{VENUS}-hph (Gsaller et al. 2014) was assembled with a SV40 NLS-venus fusion gene derived from pVenus-NLS (Furukawa et al. 2020) using DNA assembly. The selection-free expression cassette was amplified using the pair of primers P5 and P6, and the expression cassette with hygromycin resistance marker was amplified using the pair of primers P7 and P6, respectively. **(b)** Targeted integration of the NLS-venus expression constructs into the *aft4* locus using the CRISPR-Cas9 mediated transformation. Each NLS-venus expression cassette contains 50-bp of the homology arms to the corresponding guide RNAs (gRNA1 and gRNA2) to facilitate targeted integration at the *aft4* locus. The expression constructs were introduced

to *A. fumigatus* MFIG001 by a CRISPR-Cas9 mediated genome-editing system. **(c)** PCR validation of the NLS-venus expressing mutants. Correct integration of the expression cassette was verified by PCR using the primer pairs venus-Sc Fw and Rv (for integration of *NLS-venus* fusion gene), and aft4-Sc Fw and Rv (for homologous integration). The primers used in the validation PCR are shown in **(b)** and Supplementary Table 1.

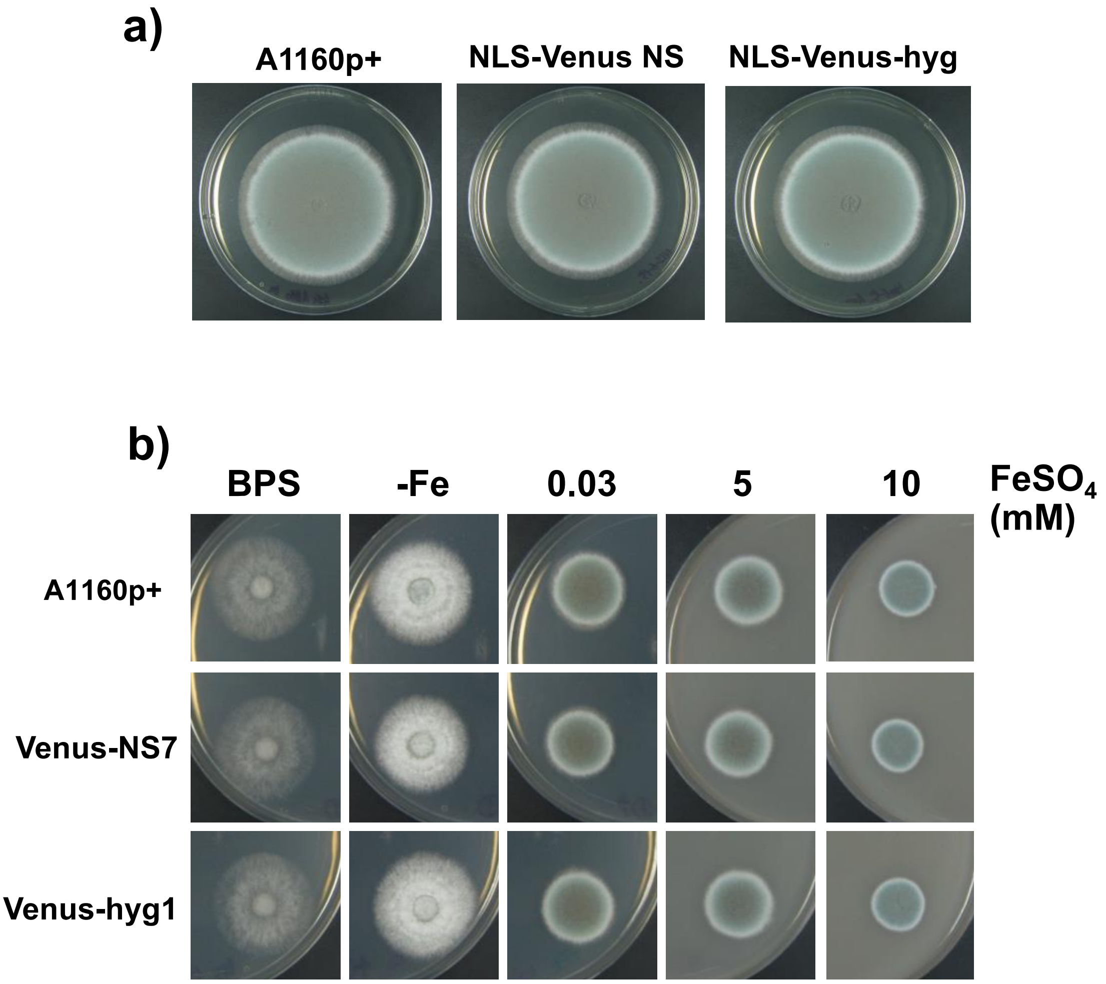

Supplementary Figure 5: Growth characteristics of the NLS-Venus expressing strains on (a) ACM and (b) AMM with different iron conditions. 1x10^4 spores were

inoculated and the plates were incubated for 72 h at 37˚C. -Fe media contains no iron, and BPS media contains 0.2 mM of the ferrous iron specific chelator bathophenanthroline disulfonate.

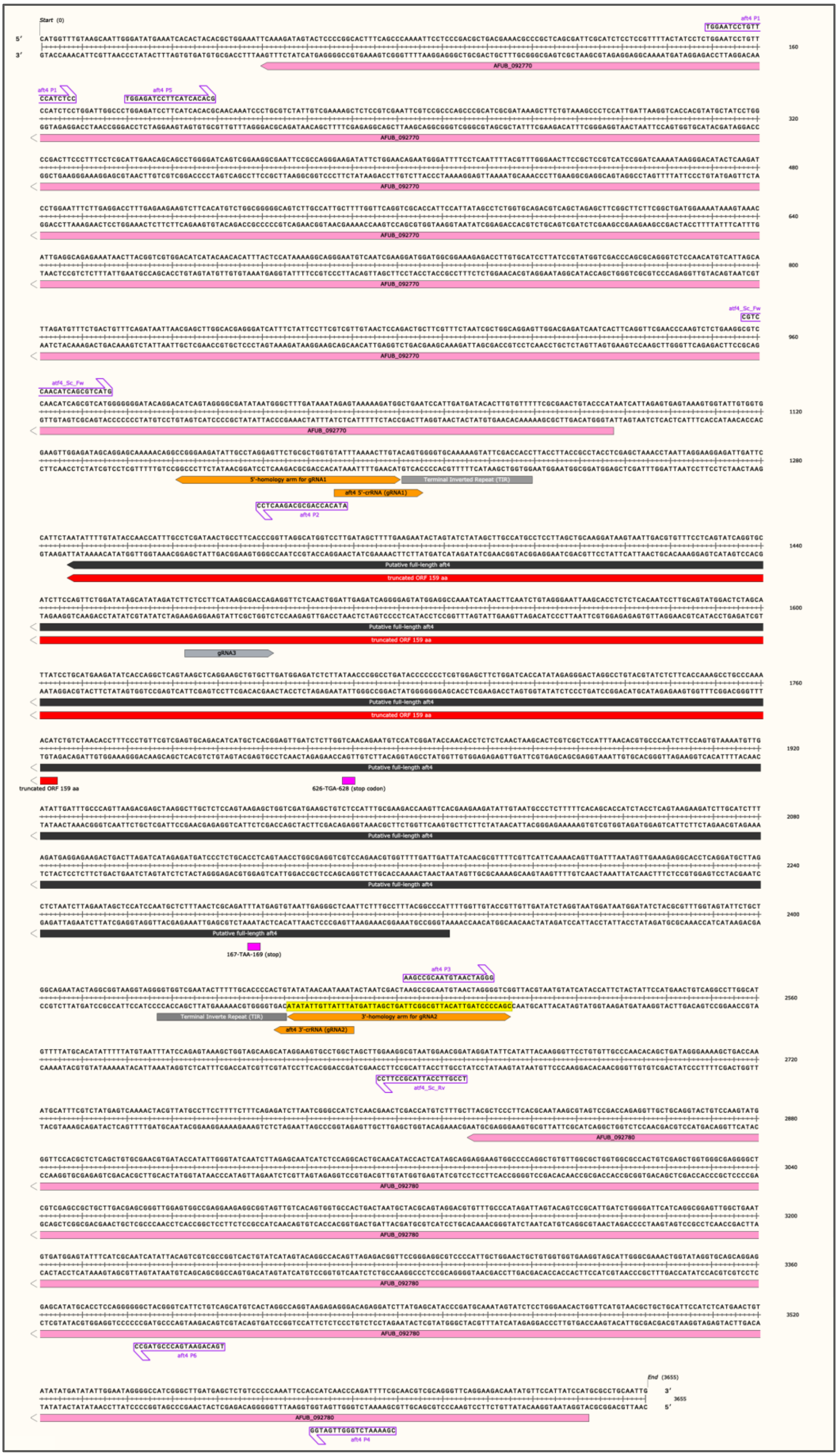
Start (0)
aft4 P1
AFUB_092770
aft4 P5
aft4_Sc_Fw
5'-homology arm for gRNA1
Terminal Inverted Repeat (TIR)
aft4 5'-crRNA (gRNA1)
aft4 P2
Putative full-length aft4
truncated ORF 159 aa
gRNA3
626-TGA-628 (stop codon)
167-TAA-169 (stop)
aft4 P3
Terminal Inverte Repeat (TIR)
3'-homology arm for gRNA2
aft4 3'-crRNA (gRNA2)
aft4_Sc_Rv
AFUB_092780
aft4 P6
End (3655)
aft4 P4

Supplementary Figure 6. Primers and gRNAs used for genome editing at the *aft4* locus. The guide RNAs, the homology arms used to target the *aft4* locus, and the primers used to construct the NLS-venus expressing mutants are shown. Nucleotide sequence of the guide RNAs and the primers is also available in Supplementary Table 1.

Supplementary Table 1. Oligonucle

Oligo name
**(a) Construction of the *aft4* -hyg knockin**
aft4 P1
aft4 P2
aft4 P3
aft4 P4
aft4 P5
aft4 P6
hph Fw
hph Rv
hph probe Fw
hph probe Rv
**(b) amplification of the aft4 *locus***
**Name**
aft4_100bp_Fw
aft4_100bp_RV
aft4 Sc Fw
atf4 Sc Rv
aft4_qPCR2_Rv
aft4_qPCR3_Fw
**(c) Generation of NLS-Venus expressing**
phapXVenus-hph inverse Fw P1
phapXVenus-hph inverse Rv P2
NLS-Venus Fw P3
NLS-Venus Rv P4
hapXP-NLS-Venus P5
hapXP-NLS-Venus P6
Hyg-hapXP-NLS-Venus P7
atf4 5’-crRNA (gRNA1)
atf4 3’-crRNA (gRNA2)
aft4 Sc Fw
atf4 Sc Rv
NLS-venus Seq1
NLS-venus Seq2
NLS-venus Seq3
NLS-venus Seq4
NLS-venus Seq5
NLS-venus Seq6
NLS-venus Seq7
**(d) qPCR analysis of the NLS-Venus expr**
venus qPCR1 Fw
venus_qPCR1 Rv
RTgpdA-FW

RTgpdA-RV
AFUB_092770 qPCR Fw
AFUB_092770 qPCR Rv
AFUB_092780 qPCR Fw
AFUB_092780 qPCR Rv

**Optional (discuss)**
hapX_qPCR1_Fw
hapX_qPCR1_Rv

atf4_crRNA_273

eotides used in this study

Sequence (5'-3')
**mutant and Southern blotting analysis**
TGGAATCCTGTTCCATCTCC
TAGTTCTGTTACCGAGCCGGATACACCAGCGCAGAACTCC
GCTCTGAACGATATGCTCCAACAAGCCGCAATGTAACTAGGG
CGAAAATCTGGGTTGATGG
TGGAGATCCTTCATCACACG
TGACAGAATGACCCGTAGCC
CCGGCTCGGTAACAGAACTAACGGCGTAACCAAAAGTCAC
GTTGGAGCATATCGTTCAGAGCTCTTGACGACCGTTGATCTG
GCCCCTACAACGACCATCAA
CCGGGTACTCGCTCTACCTA
**5'-3'**
GAGTGAGTAAAGTGGTATTGTGG
AACATGCCAAGGCCTGAC
CGTCCAACATCAGCGTCATG
TCCGTTCCATTACGCCTTCC
AAAACCACGTTCTGGACGAC
TCAGGTGCATCTTCCAGTTC
**mutants and qPCR**
TTTATCGCATCTCTGCTTG
GATTACGGATGATGAGAC
GAGTCTCATCATCCGTAATCATGGATAAAGCGGAATTAATTC
ACAAGCAGAGATGCGATAAACTACTTGTACAGCTCGTC
CCGGGAAGATATTGCCTAGGAGTTCTGCGCTGGTGTATTTAAAACTTGTATGGAGTTCCGA
CGACCCCTAGTTACATTGCGGCTTAGTCGATTAGTATTTATTGTTATATAATCCCGCTTCTTT
CCGGGAAGATATTGCCTAGGAGTTCTGCGCTGGTGTATTTAAAACTTGTAGCATGCCATTA
TATTTAAAACTTGTACAGTG
ATTTATTGTTATATACAGTG
CGTCCAACATCAGCGTCATG
TCCGTTCCATTACGCCTTCC
TCCGTAACACCCAATACGCC
CGAAGCAGCCTTTGAATGGG
TTAGCTCCAGTTCCCTTGCC
ACCCTGACCACATGAAGCAG
ACCACTACCAGCAGAACACC
CTGGTCGAAATCTCGCGTTG
GTCAACCGGAATCGCTAACG
**ressing mutants**
AGGACGACGGCAACTACAAG
AAGTCGATGCCCTTCAGCTC
GAGCTCAAAAACATCCTCGGC

CGAAGTTGGGGTTGAGGGAG
GTTGTATGATGTCCACGAC
GAGGACCTTTGAGAAGAAG
GAACCAGTGTTCCCAGGAGA
TTGGGCGAAACTGGTATAGG

AAGCCAGCCATCTCACCTTC
CGGTTTCCTGCCTGGTTTTG

TCTCCTTCATAAGCGACCAG

Note
Amplification of *hph* probe
Amplification of *hph* probe
**Target**
aft4-100bp up
aft4-100bp down
PCR validation of the NLS-venus expressing strains
PCR validation of the NLS-venus expressing strains
183 bp with intron
short 159aa ORF (C-teminal part of the ORF)
Inverse PCR of phapX^{VENUS}-hph
Inverse PCR of phapX^{VENUS}-hph
Amplification of NLS-venus
Amplification of NLS-venus
Amplification of NLS-venus cassette 1
Amplification of NLS-venus cassette 1 and 2
Amplification of NLS-venus cassette 2
crRNA for CRISPR-Cas9 transformation
crRNA for CRISPR-Cas9 transformation
PCR validation of the NLS-venus expressing strains
PCR validation of the NLS-venus expressing strains
Sanger sequencing of the NLS-venus expressing strains
Sanger sequencing of the NLS-venus expressing strains
Sanger sequencing of the NLS-venus expressing strains
Sanger sequencing of the NLS-venus expressing strains
Sanger sequencing of the NLS-venus expressing strains
Sanger sequencing of the NLS-venus expressing strains
Sanger sequencing of the NLS-venus expressing strains
qPCR of *venus*
qPCR of *venus*
qPCR of *gpdA*

qPCR of *gpdA*
qPCR of AFUB_092770
qPCR of AFUB_092770
qPCR of AFUB_092780
qPCR of AFUB_092780

qPCR of *hapX*
qPCR of *hapX*

crRNA for CRISPR-Cas9 transformation (insertion)

Supplementary Table2. Characteristic features of the

Name of safe-haven	Genomi
	**Af293**
SH1	Chr1: 1,066,076-1,068,189
SH2	Chr2: 2,196,634-2,199,345
aft4	Chr6:2,227,032-2,228,286

*Pham T, Xie X, Lin X. An intergenic "safe haven" regio

identified genetic safe-haven sites in *A. fumigatus* **.**

ic coordinate	Origin
**A1163**	
DS499594:1138672-1,140,783	Intergenic region
DS499595: 2,181,221-2,183,942	Intergenic region
DS499601: 1,810,829-1,812,208	an inactived transpson

n in Aspergillus fumigatus. Med Mycol. 2020 Nov 10;58(8):1178-1186.

Expressed genes	Virulence model tested
mNeonGreen, Hygromycin B resistance gene from E	Galleria mellonella model
	Galleria mellonella model
Hygromycin B resistance gene from *E. coli*	leukopenic mouse model
Venus fluorescent protein fused with a NLS	

doi: 10.1093/mmy/myaa009. PMID: 32171003.

Reference
Pham et al., 2020
Pham et al., 2020
This study

Click here to access/download;Table;STable 3.xlsx

alignment_overview

Isolate	TIR total	Full TIRs	Partial TIRs	aft4 Homologs	Flanking TIRs	TIR-aft4-TIR
ARAF00	4	4	0	1	2	1
ARAF00	6	6	0	2	3	2
ARAF00	6	6	0	2	3	2
ARAF00	0	0	0	0	0	0
ARAF00	2	2	0	2	1	1
ARAF00	0	0	0	0	0	0
C100	2	2	0	1	1	1
C103	2	2	0	1	1	1
C104	2	2	0	1	1	1
C105	0	0	0	0	0	0
C106	1	1	0	1	0	0
C107	2	2	0	1	1	1
C108	2	2	0	1	1	1
C109	2	2	0	1	1	1
C10	2	2	0	1	1	1
C110	2	2	0	1	1	1
C111	2	2	0	1	1	1
C112	2	2	0	1	1	1
C113	0	0	0	0	0	0
C114	2	2	0	1	1	1
C115	2	2	0	1	1	1
C117	2	2	0	1	1	1
C118	2	2	0	1	1	1
C119	2	2	0	1	1	1
C11	0	0	0	0	0	0
C120	2	2	0	1	1	1
C121	0	0	0	0	0	0
C122	2	2	0	1	1	1
C123	2	2	0	1	1	1
C124	2	2	0	1	1	1
C125	2	2	0	1	1	1
C126	2	2	0	1	1	1
C127	2	2	0	1	1	1
C128	6	6	0	2	3	2
C129	2	2	0	1	1	1
C12	2	2	0	1	1	1
C130	0	0	0	0	0	0
C131	0	0	0	0	0	0
C132	0	0	0	0	0	0
C133	2	2	0	1	1	1
C134	2	2	0	1	1	1
C135	0	0	0	0	0	0
C136	6	6	0	2	3	2
C137	6	6	0	2	3	2
C138	2	2	0	1	1	1
C139	2	2	0	1	1	1
C13	0	0	0	0	0	0
C140	2	2	0	1	1	1
C141	2	2	0	1	1	1
C142	2	2	0	1	1	1
C143	2	2	0	1	1	1
C144	4	4	0	1	2	1
C145	2	2	0	1	1	1
C146	6	6	0	2	3	2

C147	6	6	0	2	3	2
C148	6	6	0	2	3	2
C149	6	6	0	2	3	2
C14	2	2	0	1	1	1
C150	6	6	0	2	3	2
C151	6	6	0	2	3	2
C152	6	6	0	2	3	2
C153	6	6	0	2	3	2
C154	6	6	0	2	3	2
C155	2	2	0	1	1	1
C156	2	2	0	1	1	1
C157	2	2	0	1	1	1
C158	2	2	0	1	1	1
C159	2	2	0	0	1	0
C15	2	2	0	1	1	1
C160	2	2	0	0	1	0
C161	2	2	0	1	1	1
C162	0	0	0	0	0	0
C163	0	0	0	0	0	0
C164	2	2	0	3	1	1
C165	2	2	0	1	1	1
C166	0	0	0	1	0	0
C167	2	2	0	1	1	1
C168	2	2	0	1	1	1
C169	0	0	0	0	0	0
C16	2	2	0	1	1	1
C170	0	0	0	0	0	0
C171	6	6	0	2	3	2
C172	2	2	0	1	1	1
C173	0	0	0	0	0	0
C174	0	0	0	0	0	0
C175	2	2	0	1	1	1
C176	2	2	0	1	1	1
C177	0	0	0	0	0	0
C178	2	2	0	2	1	1
C179	2	2	0	1	1	1
C17	2	2	0	1	1	1
C180	2	2	0	1	1	1
C181	2	2	0	1	1	1
C182	2	2	0	1	1	1
C183	2	2	0	1	1	1
C184	6	6	0	2	3	2
C185	2	2	0	2	1	1
C186	0	0	0	0	0	0
C187	2	2	0	1	1	1
C188	0	0	0	0	0	0
C189	1	1	0	1	0	0
C18	2	2	0	1	1	1
C190	2	2	0	1	1	1
C191	0	0	0	0	0	0
C199	2	2	0	1	1	1
C19	0	0	0	0	0	0
C1	2	2	0	1	1	1
C20	2	2	0	1	1	1
C21	2	2	0	1	1	1

C220	0	0	0	0	0	0
C221	0	0	0	0	0	0
C222	0	0	0	0	0	0
C223	0	0	0	0	0	0
C22	2	2	0	1	1	1
C23	2	2	0	1	1	1
C246	0	0	0	0	0	0
C24	2	2	0	1	1	1
C25	0	0	0	0	0	0
C26	0	0	0	0	0	0
C272	2	2	0	3	1	1
C275	0	0	0	0	0	0
C27	2	2	0	1	1	1
C28	0	0	0	0	0	0
C29	2	2	0	1	1	1
C2	1	1	0	1	0	0
C30	2	2	0	1	1	1
C31	2	2	0	1	1	1
C32	2	2	0	1	1	1
C33	2	2	0	1	1	1
C341	4	4	0	2	2	2
C342	2	2	0	1	1	1
C343	2	2	0	1	1	1
C344	2	2	0	1	1	1
C345	2	2	0	1	1	1
C346	2	2	0	1	1	1
C34	0	0	0	0	0	0
C354	2	2	0	1	1	1
C355	2	2	0	1	1	1
C356	2	2	0	1	1	1
C357	2	2	0	1	1	1
C358	0	0	0	0	0	0
C359	2	2	0	1	1	1
C35	0	0	0	0	0	0
C360	0	0	0	0	0	0
C361	2	2	0	1	1	1
C362	2	2	0	1	1	1
C363	2	2	0	1	1	1
C364	2	2	0	1	1	1
C365	1	1	0	1	0	0
C366	2	2	0	1	1	1
C367	0	0	0	0	0	0
C368	2	2	0	1	1	1
C369	2	2	0	1	1	1
C36	0	0	0	0	0	0
C37	2	2	0	1	1	1
C38	0	0	0	0	0	0
C39	0	0	0	0	0	0
C3	2	2	0	1	1	1
C40	2	2	0	1	1	1
C41	2	2	0	1	1	1
C42	0	0	0	0	0	0
C43	0	0	0	0	0	0
C44	0	0	0	0	0	0
C45	2	2	0	1	1	1

C46	0	0	0	0	0	0
C47	0	0	0	0	0	0
C48	0	0	0	0	0	0
C49	2	2	0	1	1	1
C4	0	0	0	0	0	0
C50	2	2	0	1	1	1
C51	2	2	0	1	1	1
C52	2	2	0	1	1	1
C53	2	2	0	1	1	1
C54	2	2	0	1	1	1
C55	2	2	0	1	1	1
C56	2	2	0	1	1	1
C57	2	2	0	1	1	1
C58	2	2	0	1	1	1
C59	2	2	0	1	1	1
C5	2	2	0	1	1	1
C60	2	2	0	1	1	1
C61	2	2	0	1	1	1
C62	2	2	0	1	1	1
C63	2	2	0	1	1	1
C64	0	0	0	0	0	0
C65	0	0	0	0	0	0
C66	0	0	0	0	0	0
C67	2	2	0	1	1	1
C68	2	2	0	1	1	1
C69	2	2	0	1	1	1
C6	2	2	0	1	1	1
C70	2	2	0	1	1	1
C71	2	2	0	1	1	1
C72	2	2	0	1	1	1
C73	2	2	0	1	1	1
C74	2	2	0	1	1	1
C75	0	0	0	0	0	0
C76	0	0	0	0	0	0
C77	0	0	0	0	0	0
C78	2	2	0	1	1	1
C79	0	0	0	0	0	0
C7	0	0	0	0	0	0
C80	2	2	0	1	1	1
C81	2	2	0	1	1	1
C82	0	0	0	0	0	0
C83	2	2	0	1	1	1
C84	2	2	0	1	1	1
C85	2	2	0	1	1	1
C86	2	2	0	1	1	1
C87	0	0	0	0	0	0
C88	2	2	0	1	1	1
C89	0	0	0	0	0	0
C8	2	2	0	1	1	1
C91	0	0	0	0	0	0
C92	1	1	0	1	0	0
C93	0	0	0	0	0	0
C95	0	0	0	1	0	0
C96	0	0	0	0	0	0

aft4 with no flanks	Flanking TIRs minus TIR-aft4-TIR	TIR-uORF-TIR	TIR-uORF-TIR single strand
0	1	0	0
0	1	0	0
0	1	0	0
0	0	0	0
1	0	0	0
0	0	0	0
0	0	0	0
0	0	0	0
0	0	0	0
0	0	0	0
1	0	0	0
0	0	0	0
0	0	0	0
0	0	0	0
0	0	0	0
0	0	0	0
0	0	0	0
0	0	0	0
0	0	0	0
0	0	0	0
0	0	0	0
0	0	0	0
0	0	0	0
0	0	0	0
0	0	0	0
0	0	0	0
0	0	0	0
0	0	0	0
0	0	0	0
0	0	0	0
0	0	0	0
0	0	0	0
0	0	0	0
0	1	0	0
0	0	0	0
0	0	0	0
0	0	0	0
0	0	0	0
0	0	0	0
0	0	0	0
0	0	0	0
0	0	0	0
0	1	0	0
0	1	0	0
0	0	0	0
0	0	0	0
0	0	0	0
0	0	0	0
0	0	0	0
0	0	0	0
0	0	0	0
0	1	0	0
0	0	0	0
0	1	0	0

0	1	0	0
0	1	0	0
0	1	0	0
0	0	0	0
0	1	0	0
0	1	0	0
0	1	0	0
0	1	0	0
0	1	0	0
0	0	0	0
0	0	0	0
0	0	0	0
0	0	0	0
0	1	0	0
0	0	0	0
0	1	0	0
0	0	0	0
0	0	0	0
0	0	0	0
2	0	0	0
0	0	0	0
1	0	0	0
0	0	0	0
0	0	0	0
0	0	0	0
0	0	0	0
0	0	0	0
0	1	0	0
0	0	0	0
0	0	0	0
0	0	0	0
0	0	0	0
0	0	0	0
0	0	0	0
1	0	0	0
0	0	0	0
0	0	0	0
0	0	0	0
0	0	0	0
0	0	0	0
0	0	0	0
0	1	0	0
1	0	0	0
0	0	0	0
0	0	0	0
0	0	0	0
1	0	0	0
0	0	0	0
0	0	0	0
0	0	0	0
0	0	0	0
0	0	0	0
0	0	0	0
0	0	0	0
0	0	0	0

0	0	0	0
0	0	0	0
0	0	0	0
0	0	0	0
0	0	0	0
0	0	0	0
0	0	0	0
0	0	0	0
0	0	0	0
0	0	0	0
2	0	0	0
0	0	0	0
0	0	0	0
0	0	0	0
0	0	0	0
1	0	0	0
0	0	0	0
0	0	0	0
0	0	0	0
0	0	0	0
0	0	0	0
0	0	0	0
0	0	0	0
0	0	0	0
0	0	0	0
0	0	0	0
0	0	0	0
0	0	0	0
0	0	0	0
0	0	0	0
0	0	0	0
0	0	0	0
0	0	0	0
0	0	0	0
0	0	0	0
0	0	0	0
0	0	0	0
0	0	0	0
0	0	0	0
1	0	0	0
0	0	0	0
0	0	0	0
0	0	0	0
0	0	0	0
0	0	0	0
0	0	0	0
0	0	0	0
0	0	0	0
0	0	0	0
0	0	0	0
0	0	0	0
0	0	0	0
0	0	0	0
0	0	0	0
0	0	0	0

0	0	0	0
0	0	0	0
0	0	0	0
0	0	0	0
0	0	0	0
0	0	0	0
0	0	0	0
0	0	0	0
0	0	0	0
0	0	0	0
0	0	0	0
0	0	0	0
0	0	0	0
0	0	0	0
0	0	0	0
0	0	0	0
0	0	0	0
0	0	0	0
0	0	0	0
0	0	0	0
0	0	0	0
0	0	0	0
0	0	0	0
0	0	0	0
0	0	0	0
0	0	0	0
0	0	0	0
0	0	0	0
0	0	0	0
0	0	0	0
0	0	0	0
0	0	0	0
0	0	0	0
0	0	0	0
0	0	0	0
0	0	0	0
0	0	0	0
0	0	0	0
0	0	0	0
0	0	0	0
0	0	0	0
0	0	0	0
0	0	0	0
0	0	0	0
0	0	0	0
0	0	0	0
0	0	0	0
0	0	0	0
0	0	0	0
0	0	0	0
1	0	0	0
0	0	0	0
1	0	0	0
0	0	0	0

TIR-uORF-TIR double stranded	TIR-TIR (no ORF)	NH1	NH2	NH3
0	1	1	0	0
0	1	1	1	0
0	1	1	1	0
0	0	0	0	0
0	0	1	0	0
0	0	0	0	0
0	0	1	0	0
0	0	1	0	0
0	0	1	0	0
0	0	0	0	0
0	0	0	0	0
0	0	1	0	0
0	0	1	0	0
0	0	1	0	0
0	0	1	0	0
0	0	1	0	0
0	0	1	0	0
0	0	1	0	0
0	0	0	0	0
0	0	1	0	0
0	0	1	0	0
0	0	1	0	0
0	0	1	0	0
0	0	1	0	0
0	0	0	0	0
0	0	1	0	0
0	0	0	0	0
0	0	1	0	0
0	0	1	0	0
0	0	1	0	0
0	0	1	0	0
0	0	1	0	0
0	0	1	0	0
0	1	1	1	0
0	0	1	0	0
0	0	1	0	0
0	0	0	0	0
0	0	0	0	0
0	0	0	0	0
0	0	1	0	0
0	0	1	0	0
0	0	0	0	0
0	1	1	1	0
0	1	1	1	0
0	0	1	0	0
0	0	1	0	0
0	0	0	0	0
0	0	1	0	0
0	0	1	0	0
0	0	1	0	0
0	0	1	0	0
0	1	1	0	0
0	0	1	0	0
0	1	1	1	0

0	1	1	1	0
0	1	1	1	0
0	1	1	1	0
0	0	1	0	0
0	1	1	1	0
0	1	1	1	0
0	1	1	1	0
0	1	1	1	0
0	1	1	1	0
0	0	1	0	0
0	0	1	0	0
0	0	1	0	0
0	0	1	0	0
0	1	0	0	0
0	0	1	0	0
0	1	0	0	0
0	0	1	0	0
0	0	0	0	0
0	0	0	0	0
0	0	1	0	0
0	0	1	0	0
0	0	0	0	0
0	0	1	0	0
0	0	1	0	0
0	0	0	0	0
0	0	1	0	0
0	0	0	0	0
0	1	1	1	0
0	0	1	0	0
0	0	0	0	0
0	0	0	0	0
0	0	1	0	0
0	0	1	0	0
0	0	0	0	0
0	0	1	0	0
0	0	1	0	0
0	0	1	0	0
0	0	1	0	0
0	0	1	0	0
0	0	1	0	0
0	0	1	0	0
0	1	1	0	1
0	0	1	0	0
0	0	0	0	0
0	0	1	0	0
0	0	0	0	0
0	0	0	0	0
0	0	1	0	0
0	0	1	0	0
0	0	0	0	0
0	0	1	0	0
0	0	0	0	0
0	0	1	0	0
0	0	1	0	0
0	0	1	0	0

0	0	0	0	0
0	0	0	0	0
0	0	0	0	0
0	0	0	0	0
0	0	1	0	0
0	0	1	0	0
0	0	0	0	0
0	0	1	0	0
0	0	0	0	0
0	0	0	0	0
0	0	1	0	0
0	0	0	0	0
0	0	1	0	0
0	0	0	0	0
0	0	1	0	0
0	0	0	0	0
0	0	1	0	0
0	0	1	0	0
0	0	1	0	0
0	0	1	0	0
0	0	1	1	0
0	0	1	0	0
0	0	1	0	0
0	0	1	0	0
0	0	1	0	0
0	0	1	0	0
0	0	0	0	0
0	0	1	0	0
0	0	1	0	0
0	0	1	0	0
0	0	1	0	0
0	0	0	0	0
0	0	1	0	0
0	0	0	0	0
0	0	0	0	0
0	0	1	0	0
0	0	1	0	0
0	0	1	0	0
0	0	1	0	0
0	0	0	0	0
0	0	1	0	0
0	0	0	0	0
0	0	1	0	0
0	0	1	0	0
0	0	0	0	0
0	0	1	0	0
0	0	0	0	0
0	0	0	0	0
0	0	1	0	0
0	0	1	0	0
0	0	1	0	0
0	0	0	0	0
0	0	0	0	0
0	0	0	0	0
0	0	1	0	0

0	0	0	0	0
0	0	0	0	0
0	0	0	0	0
0	0	1	0	0
0	0	0	0	0
0	0	1	0	0
0	0	1	0	0
0	0	1	0	0
0	0	1	0	0
0	0	1	0	0
0	0	1	0	0
0	0	1	0	0
0	0	1	0	0
0	0	1	0	0
0	0	1	0	0
0	0	1	0	0
0	0	1	0	0
0	0	1	0	0
0	0	1	0	0
0	0	1	0	0
0	0	0	0	0
0	0	0	0	0
0	0	0	0	0
0	0	1	0	0
0	0	1	0	0
0	0	1	0	0
0	0	1	0	0
0	0	1	0	0
0	0	1	0	0
0	0	1	0	0
0	0	1	0	0
0	0	1	0	0
0	0	0	0	0
0	0	0	0	0
0	0	0	0	0
0	0	1	0	0
0	0	0	0	0
0	0	0	0	0
0	0	1	0	0
0	0	1	0	0
0	0	0	0	0
0	0	1	0	0
0	0	1	0	0
0	0	1	0	0
0	0	1	0	0
0	0	0	0	0
0	0	1	0	0
0	0	0	0	0
0	0	1	0	0
0	0	0	0	0
0	0	0	0	0
0	0	0	0	0
0	0	0	0	0
0	0	0	0	0

stop

genome	R15	Q103	R119	S168	R182
ARAF001_NH1_k119_1121:5262-6307(+)	1	1	1	1	1
ARAF002_NH1_k119_2094:64203-65248(-)	1	1	1	1	1
C104_NH1_k141_1207:5233-6278(+)	1	1	1	1	1
C107_NH1_k141_258:11182-12227(-)	1	1	1	1	1
C114_NH1_k141_382:11198-12243(-)	1	1	1	1	1
C115_NH1_k141_937:11148-12193(-)	1	1	1	1	1
C122_NH1_k141_157:5223-6268(+)	1	1	1	1	1
C12_NH1_k141_605:11159-12204(-)	1	1	1	1	1
C144_NH1_k141_374:5251-6296(+)	1	1	1	1	1
C149_NH1_k141_1298:10987-12032(-)	1	1	1	1	1
C150_NH1_k141_851:11029-12074(-)	1	1	1	1	1
C151_NH1_k141_747:11128-12173(-)	1	1	1	1	1
C161_NH1_k141_719:10684-11729(-)	1	1	1	1	1
C171_NH1_k141_2101:5262-6307(+)	1	1	1	1	1
C341_NH1_k141_398:103576-104621(+)	1	1	1	1	1
C354_NH1_k141_549:61621-62666(-)	1	1	1	1	1
C78_NH1_k141_891:11197-12242(-)	1	1	1	1	0
C355_NH1_k141_552:61927-62972(-)	1	1	0	1	0
C357_NH1_k141_637:61997-63042(-)	1	1	0	1	0
ARAF003_NH1_k119_1519:5258-6303(+)	0	0	0	1	0
ARAF005_NH1_k119_1110:19503-20548(-)	1	0	0	1	0
C100_NH1_k141_617:10998-12043(-)	1	0	0	1	0
C103_NH1_k141_1788:5244-6289(+)	1	0	0	1	0
C108_NH1_k141_570:11166-12211(-)	1	0	0	1	0
C109_NH1_k141_608:10853-11898(-)	1	0	0	1	0
C10_NH1_k141_608:5247-6292(+)	1	0	0	1	0
C110_NH1_k141_1428:6555-7600(-)	1	0	0	1	0
C111_NH1_k141_825:5240-6285(+)	1	0	0	1	0
C112_NH1_k141_1573:11168-12213(-)	1	0	0	1	0
C117_NH1_k141_625:11178-12223(-)	1	0	0	1	0
C118_NH1_k141_540:5262-6307(+)	1	0	0	1	0
C119_NH1_k141_1690:10836-11881(-)	1	0	0	1	0
C120_NH1_k141_735:6489-7534(-)	1	0	0	1	0
C123_NH1_k141_834:5237-6282(+)	1	0	0	1	0
C124_NH1_k141_1454:10709-11754(-)	1	0	0	1	0
C125_NH1_k141_2115:6705-7750(-)	1	0	0	1	0
C126_NH1_k141_92:17210-18255(-)	1	0	0	1	0
C127_NH1_k141_821:10877-11922(-)	1	0	0	1	0
C128_NH1_k141_1129:5245-6290(+)	1	0	0	1	0
C129_NH1_k141_1741:11202-12247(-)	1	0	0	1	0
C133_NH1_k141_1470:5246-6291(+)	1	0	0	1	0
C134_NH1_k141_521:10981-12026(-)	1	0	0	1	0
C136_NH1_k141_1285:11026-12071(-)	1	0	0	1	0
C137_NH1_k141_1038:5183-6228(+)	1	0	0	1	0
C138_NH1_k141_434:2609-3654(-)	1	0	0	1	0
C139_NH1_k141_1028:10975-12020(-)	1	0	0	1	0
C140_NH1_k141_521:5248-6293(+)	1	0	0	1	0
C141_NH1_k141_1340:5246-6291(+)	1	0	0	1	0
C142_NH1_k141_953:24754-25799(-)	1	0	0	1	0
C143_NH1_k141_304:5246-6291(+)	1	0	0	1	0
C145_NH1_k141_1364:10986-12031(-)	1	0	0	1	0
C146_NH1_k141_134:10790-11835(-)	0	0	0	1	0
C147_NH1_k141_1389:10762-11807(-)	0	0	0	1	0
C148_NH1_k141_1243:10785-11830(-)	0	0	0	1	0

C14_NH1_k141_6:5243-6288(+)	1	0	0	1	0
C152_NH1_k141_1469:10730-11775(-)	0	0	0	1	0
C153_NH1_k141_954:5238-6283(+)	0	0	0	1	0
C154_NH1_k141_376:10761-11806(-)	0	0	0	1	0
C155_NH1_k141_1295:10985-12030(-)	1	0	0	1	0
C156_NH1_k141_613:11035-12080(-)	1	0	0	1	0
C157_NH1_k141_685:5245-6290(+)	1	0	0	1	0
C158_NH1_k141_1037:5227-6272(+)	1	0	0	1	0
C15_NH1_k141_139:11846-12891(+)	1	0	0	1	0
C164_NH1_k141_485:11202-12247(-)	1	0	0	1	0
C165_NH1_k141_141:10999-12044(-)	1	0	0	1	0
C167_NH1_k141_429:2609-3654(-)	1	0	0	1	0
C168_NH1_k141_798:11171-12216(-)	1	0	0	1	0
C16_NH1_k141_1419:5241-6286(+)	1	0	0	1	0
C172_NH1_k141_297:6386-7431(-)	1	0	0	1	0
C175_NH1_k141_432:5265-6310(+)	1	0	0	1	0
C176_NH1_k141_747:5254-6299(+)	1	0	0	1	0
C178_NH1_k141_2432:6525-7570(-)	1	0	0	1	0
C179_NH1_k141_1333:11201-12246(-)	1	0	0	1	0
C17_NH1_k141_550:5240-6285(+)	1	0	0	1	0
C180_NH1_k141_782:5227-6272(+)	1	0	0	1	0
C181_NH1_k141_527:5224-6269(+)	1	0	0	1	0
C182_NH1_k141_266:5240-6285(+)	1	0	0	1	0
C183_NH1_k141_1175:11152-12197(-)	1	0	0	1	0
C184_NH1_k141_528:10889-11934(-)	1	0	0	1	0
C185_NH1_k141_144:10857-11902(-)	1	0	0	1	0
C187_NH1_k141_152:10990-12035(-)	1	0	0	1	0
C18_NH1_k141_1001:5246-6291(+)	1	0	0	1	0
C190_NH1_k141_370:2609-3654(-)	1	0	0	1	0
C199_NH1_k141_836:5173-6218(+)	1	0	0	1	0
C1_NH1_k141_529:5246-6291(+)	1	0	0	1	0
C20_NH1_k141_1379:5239-6284(+)	1	0	0	1	0
C21_NH1_k141_899:5237-6282(+)	1	0	0	1	0
C22_NH1_k141_740:11063-12108(-)	1	0	0	1	0
C23_NH1_k141_1176:5236-6281(+)	1	0	0	1	0
C24_NH1_k141_1268:6457-7502(-)	1	0	0	1	0
C272_NH1_k141_319:5184-6229(+)	1	0	0	1	0
C27_NH1_k141_1336:11168-12213(-)	1	0	0	1	0
C29_NH1_k141_793:5239-6284(+)	1	0	0	1	0
C30_NH1_k141_4485:10328-11373(-)	1	0	0	1	0
C31_NH1_k141_1085:5237-6282(+)	1	0	0	1	0
C32_NH1_k141_936:5247-6292(+)	1	0	0	1	0
C33_NH1_k141_823:11170-12215(-)	1	0	0	1	0
C342_NH1_k141_665:52306-53351(+)	1	0	0	1	0
C343_NH1_k141_73:85716-86761(-)	1	0	0	1	0
C344_NH1_k141_298:120532-121577(+)	1	0	0	1	0
C345_NH1_k141_743:63671-64716(-)	1	0	0	1	0
C346_NH1_k141_130:22919-23964(+)	1	0	0	1	0
C356_NH1_k141_482:52304-53349(+)	1	0	0	1	0
C359_NH1_k141_352:74873-75918(-)	1	0	0	1	0
C361_NH1_k141_77:8860-9905(+)	1	0	0	1	0
C362_NH1_k141_276:44624-45669(-)	1	0	0	1	0
C363_NH1_k141_87:12875-13920(+)	1	0	0	1	0
C364_NH1_k141_724:76787-77832(-)	1	0	0	1	0
C366_NH1_k141_123:23953-24998(+)	1	0	0	1	0

C368_NH1_k141_1027:45287-46332(-)	1	0	0	1	0
C369_NH1_k141_361:24566-25611(+)	1	0	0	1	0
C37_NH1_k141_1251:11164-12209(-)	1	0	0	1	0
C3_NH1_k141_1242:5147-6192(+)	1	0	0	1	0
C40_NH1_k141_146:11197-12242(-)	1	0	0	1	0
C41_NH1_k141_157:11011-12056(-)	1	0	0	1	0
C45_NH1_k141_354:5245-6290(+)	1	0	0	1	0
C49_NH1_k141_1125:5242-6287(+)	1	0	0	1	0
C50_NH1_k141_451:11183-12228(-)	1	0	0	1	0
C51_NH1_k141_225:10874-11919(-)	1	0	0	1	0
C52_NH1_k141_38:5167-6212(+)	1	0	0	1	0
C53_NH1_k141_1335:11862-12907(+)	1	0	0	1	0
C54_NH1_k141_1966:10984-12029(-)	1	0	0	1	0
C55_NH1_k141_1178:5240-6285(+)	1	0	0	1	0
C56_NH1_k141_180:11174-12219(-)	1	0	0	1	0
C57_NH1_k141_848:11204-12249(-)	1	0	0	1	0
C58_NH1_k141_1188:5241-6286(+)	1	0	0	1	0
C59_NH1_k141_231:11013-12058(-)	1	0	0	1	0
C5_NH1_k141_754:5236-6281(+)	1	0	0	1	0
C60_NH1_k141_1007:11032-12077(-)	1	0	0	1	0
C61_NH1_k141_733:5252-6297(+)	1	0	0	1	0
C62_NH1_k141_1208:5253-6298(+)	1	0	0	1	0
C63_NH1_k141_1259:5252-6297(+)	1	0	0	1	0
C67_NH1_k141_1195:5247-6292(+)	1	0	0	1	0
C68_NH1_k141_1511:11171-12216(-)	1	0	0	1	0
C69_NH1_k141_142:11173-12218(-)	1	0	0	1	0
C6_NH1_k141_467:5228-6273(+)	1	0	0	1	0
C70_NH1_k141_951:5246-6291(+)	1	0	0	1	0
C71_NH1_k141_271:5248-6293(+)	1	0	0	1	0
C72_NH1_k141_129:11173-12218(-)	1	0	0	1	0
C73_NH1_k141_745:11186-12231(-)	1	0	0	1	0
C74_NH1_k141_485:5255-6300(+)	1	0	0	1	0
C80_NH1_k141_774:5241-6286(+)	1	0	0	1	0
C81_NH1_k141_1073:11032-12077(-)	1	0	0	1	0
C83_NH1_k141_223:5245-6290(+)	1	0	0	1	0
C84_NH1_k141_1337:11179-12224(-)	1	0	0	1	0
C85_NH1_k141_354:5232-6277(+)	1	0	0	1	0
C86_NH1_k141_1033:10881-11926(-)	1	0	0	1	0
C88_NH1_k141_1397:11163-12208(-)	1	0	0	1	0
C8_NH1_k141_1452:11185-12230(-)	1	0	0	1	0

Isolate	NH1	NH2	NH3
ARAF0C	98.9	0	0
ARAF0C	98.9	91.3	0
ARAF0C	99.4	91.3	0
ARAF0C	0	0	0
ARAF0C	99.3	0	0
ARAF0C	0	0	0
C100	99.4	0	0
C103	99.4	0	0
C104	99	0	0
C105	0	0	0
C106	0	0	0
C107	98.9	0	0
C108	99.4	0	0
C109	99.4	0	0
C10	99.4	0	0
C110	99.4	0	0
C111	99.4	0	0
C112	99.4	0	0
C113	0	0	0
C114	99	0	0
C115	98.9	0	0
C117	99.4	0	0
C118	99.3	0	0
C119	99.4	0	0
C11	0	0	0
C120	99.3	0	0
C121	0	0	0
C122	98.9	0	0
C123	99.4	0	0
C124	99.4	0	0
C125	99.4	0	0
C126	99.4	0	0
C127	99.5	0	0
C128	99.4	91.3	0
C129	99.4	0	0
C12	98.9	0	0
C130	0	0	0
C131	0	0	0
C132	0	0	0
C133	99.4	0	0
C134	99.4	0	0
C135	0	0	0
C136	99.4	91.3	0
C137	99.4	91.3	0
C138	99.5	0	0
C139	99.4	0	0
C13	0	0	0
C140	99.4	0	0
C141	99.4	0	0
C142	99.4	0	0
C143	99.4	0	0
C144	98.9	0	0
C145	99.4	0	0
C146	99.4	91.3	0

C147	99.4	91.3	0
C148	99.4	91.3	0
C149	98.9	91.3	0
C14	99.4	0	0
C150	98.9	91.3	0
C151	98.9	91.3	0
C152	99.4	91.3	0
C153	99.4	91.3	0
C154	99.4	91.3	0
C155	99.4	0	0
C156	99.4	0	0
C157	99.4	0	0
C158	99.4	0	0
C159	0	0	0
C15	99.4	0	0
C160	0	0	0
C161	98.9	0	0
C162	0	0	0
C163	0	0	0
C164	99.4	0	0
C165	99.5	0	0
C166	0	0	0
C167	99.5	0	0
C168	99.4	0	0
C169	0	0	0
C16	99.4	0	0
C170	0	0	0
C171	98.9	91.3	0
C172	99.4	0	0
C173	0	0	0
C174	0	0	0
C175	99.4	0	0
C176	99.4	0	0
C177	0	0	0
C178	99.3	0	0
C179	99.4	0	0
C17	99.4	0	0
C180	99.5	0	0
C181	99.5	0	0
C182	99.4	0	0
C183	99.5	0	0
C184	99.4	0	94.2
C185	99.4	0	0
C186	0	0	0
C187	99.4	0	0
C188	0	0	0
C189	0	0	0
C18	99.4	0	0
C190	99.5	0	0
C191	0	0	0
C199	99.4	0	0
C19	0	0	0
C1	99.4	0	0
C20	99.4	0	0
C21	99.4	0	0

C220	0	0	0
C221	0	0	0
C222	0	0	0
C223	0	0	0
C22	99.4	0	0
C23	99.4	0	0
C246	0	0	0
C24	99.4	0	0
C25	0	0	0
C26	0	0	0
C272	99.4	0	0
C275	0	0	0
C27	99.4	0	0
C28	0	0	0
C29	99.4	0	0
C2	0	0	0
C30	99.4	0	0
C31	99.4	0	0
C32	99.4	0	0
C33	99.4	0	0
C341	98.9	91.3	0
C342	99.4	0	0
C343	99.4	0	0
C344	99.5	0	0
C345	99.4	0	0
C346	99.4	0	0
C34	0	0	0
C354	98.9	0	0
C355	99.2	0	0
C356	99.4	0	0
C357	99.3	0	0
C358	0	0	0
C359	99.4	0	0
C35	0	0	0
C360	0	0	0
C361	99.3	0	0
C362	99.5	0	0
C363	99.4	0	0
C364	99.4	0	0
C365	0	0	0
C366	99.4	0	0
C367	0	0	0
C368	99.4	0	0
C369	99.4	0	0
C36	0	0	0
C37	99.5	0	0
C38	0	0	0
C39	0	0	0
C3	99.4	0	0
C40	99.5	0	0
C41	99.5	0	0
C42	0	0	0
C43	0	0	0
C44	0	0	0
C45	99.4	0	0

C46	0	0	0
C47	0	0	0
C48	0	0	0
C49	99.4	0	0
C4	0	0	0
C50	99.4	0	0
C51	99.4	0	0
C52	99.4	0	0
C53	99.4	0	0
C54	99.4	0	0
C55	99.4	0	0
C56	99.4	0	0
C57	99.4	0	0
C58	99.4	0	0
C59	99.4	0	0
C5	99.4	0	0
C60	99.4	0	0
C61	99.4	0	0
C62	99.4	0	0
C63	99.4	0	0
C64	0	0	0
C65	0	0	0
C66	0	0	0
C67	99.4	0	0
C68	99.4	0	0
C69	99.4	0	0
C6	99.4	0	0
C70	99.4	0	0
C71	99.4	0	0
C72	99.4	0	0
C73	99.4	0	0
C74	99.4	0	0
C75	0	0	0
C76	0	0	0
C77	0	0	0
C78	99	0	0
C79	0	0	0
C7	0	0	0
C80	99.4	0	0
C81	99.4	0	0
C82	0	0	0
C83	99.4	0	0
C84	99.4	0	0
C85	99.4	0	0
C86	99.5	0	0
C87	0	0	0
C88	99.4	0	0
C89	0	0	0
C8	99.4	0	0
C91	0	0	0
C92	0	0	0
C93	0	0	0
C95	0	0	0
C96	0	0	0

Isolate	NH1	NH2	NH3
ARAF00	45.4	0	0
ARAF00	45.4	44.6	0
ARAF00	47.7	44.6	0
ARAF00	0	0	0
ARAF00	45.4	0	0
ARAF00	0	0	0
C100	45.4	0	0
C103	45.4	0	0
C104	45.4	0	0
C105	0	0	0
C106	0	0	0
C107	45.4	0	0
C108	45.4	0	0
C109	45.4	0	0
C10	45.4	0	0
C110	45.4	0	0
C111	45.4	0	0
C112	45.4	0	0
C113	0	0	0
C114	45.4	0	0
C115	45.4	0	0
C117	45.4	0	0
C118	45.4	0	0
C119	45.4	0	0
C11	0	0	0
C120	45.4	0	0
C121	0	0	0
C122	45.4	0	0
C123	45.4	0	0
C124	45.4	0	0
C125	45.4	0	0
C126	45.4	0	0
C127	45.7	0	0
C128	45.4	44.6	0
C129	45.4	0	0
C12	45.4	0	0
C130	0	0	0
C131	0	0	0
C132	0	0	0
C133	45.4	0	0
C134	45.4	0	0
C135	0	0	0
C136	45.4	44.6	0
C137	45.4	44.6	0
C138	45.7	0	0
C139	45.4	0	0
C13	0	0	0
C140	45.4	0	0
C141	45.4	0	0
C142	45.4	0	0
C143	45.4	0	0
C144	45.4	0	0
C145	45.4	0	0
C146	47.7	44.6	0

C147	47.7	44.6	0
C148	47.7	44.6	0
C149	45.4	44.6	0
C14	45.4	0	0
C150	45.4	44.6	0
C151	45.4	44.6	0
C152	47.7	44.6	0
C153	47.7	44.6	0
C154	47.7	44.6	0
C155	45.4	0	0
C156	45.4	0	0
C157	45.4	0	0
C158	45.4	0	0
C159	0	0	0
C15	45.4	0	0
C160	0	0	0
C161	45.4	0	0
C162	0	0	0
C163	0	0	0
C164	45.4	0	0
C165	45.7	0	0
C166	0	0	0
C167	45.7	0	0
C168	45.4	0	0
C169	0	0	0
C16	45.4	0	0
C170	0	0	0
C171	45.4	44.6	0
C172	45.4	0	0
C173	0	0	0
C174	0	0	0
C175	45.7	0	0
C176	45.4	0	0
C177	0	0	0
C178	45.4	0	0
C179	45.4	0	0
C17	45.4	0	0
C180	45.7	0	0
C181	45.7	0	0
C182	45.4	0	0
C183	45.7	0	0
C184	45.4	0	91.4
C185	45.4	0	0
C186	0	0	0
C187	45.4	0	0
C188	0	0	0
C189	0	0	0
C18	45.4	0	0
C190	45.7	0	0
C191	0	0	0
C199	45.4	0	0
C19	0	0	0
C1	45.4	0	0
C20	45.4	0	0
C21	45.4	0	0

C220	0	0	0
C221	0	0	0
C222	0	0	0
C223	0	0	0
C22	45.4	0	0
C23	45.4	0	0
C246	0	0	0
C24	45.4	0	0
C25	0	0	0
C26	0	0	0
C272	45.4	0	0
C275	0	0	0
C27	45.4	0	0
C28	0	0	0
C29	45.4	0	0
C2	0	0	0
C30	45.4	0	0
C31	45.4	0	0
C32	45.4	0	0
C33	45.4	0	0
C341	45.4	44.6	0
C342	45.4	0	0
C343	45.4	0	0
C344	45.7	0	0
C345	45.4	0	0
C346	45.4	0	0
C34	0	0	0
C354	45.4	0	0
C355	45.4	0	0
C356	45.4	0	0
C357	45.4	0	0
C358	0	0	0
C359	45.4	0	0
C35	0	0	0
C360	0	0	0
C361	45.4	0	0
C362	45.7	0	0
C363	45.4	0	0
C364	45.4	0	0
C365	0	0	0
C366	45.4	0	0
C367	0	0	0
C368	45.4	0	0
C369	45.4	0	0
C36	0	0	0
C37	45.7	0	0
C38	0	0	0
C39	0	0	0
C3	45.4	0	0
C40	45.7	0	0
C41	45.7	0	0
C42	0	0	0
C43	0	0	0
C44	0	0	0
C45	45.4	0	0

C46	0	0	0
C47	0	0	0
C48	0	0	0
C49	45.4	0	0
C4	0	0	0
C50	45.4	0	0
C51	45.4	0	0
C52	45.4	0	0
C53	45.4	0	0
C54	45.4	0	0
C55	45.4	0	0
C56	45.4	0	0
C57	45.4	0	0
C58	45.4	0	0
C59	45.4	0	0
C5	45.4	0	0
C60	45.4	0	0
C61	45.4	0	0
C62	45.4	0	0
C63	45.4	0	0
C64	0	0	0
C65	0	0	0
C66	0	0	0
C67	45.4	0	0
C68	45.4	0	0
C69	45.4	0	0
C6	45.4	0	0
C70	45.4	0	0
C71	45.4	0	0
C72	45.4	0	0
C73	45.4	0	0
C74	45.4	0	0
C75	0	0	0
C76	0	0	0
C77	0	0	0
C78	45.4	0	0
C79	0	0	0
C7	0	0	0
C80	45.4	0	0
C81	45.4	0	0
C82	0	0	0
C83	45.4	0	0
C84	45.4	0	0
C85	45.4	0	0
C86	45.7	0	0
C87	0	0	0
C88	45.4	0	0
C89	0	0	0
C8	45.4	0	0
C91	0	0	0
C92	0	0	0
C93	0	0	0
C95	0	0	0
C96	0	0	0

Takanori Furukawa: Conceptualization, Methodology, Formal analysis, Writing – Original Draft **Norman van Rhijn:** Conceptualization, Methodology, Formal analysis, Writing – Original Draft**,** Writing – Review and Editing **Harry Chown:** Conceptualization, Methodology, Software, Formal analysis, Writing – Original Draft **Johanna Rhodes:** Conceptualization, Methodology, Software, Formal analysis **Narjes Alfuraji:** Investigation, Formal analysis **Rachael Fortune-Grant:** Investigation, Formal analysis **Elaine Bignell:** Funding acquisition, Supervision **Matthew C. Fisher:** Funding acquisition, Supervision, Conceptualization, Resources **Michael Bromley:** Conceptualization, Methodology, Formal analysis, Writing – Original Draft**,** Writing – Review and Editing, Funding acquisition, Supervision
